# Asymptomatic Nonsyndromic Multiple Supernumerary Premolars

**DOI:** 10.5005/jp-journals-10005-1141

**Published:** 2012-02-24

**Authors:** Seema Gupta, Mridula Goswami

**Affiliations:** Senior Resident, Department of Pediatric Dentistry, Maulana Azad Institute of Dental Sciences, New Delhi, India, e-mail: seema.mds@gmail.com; Professor and Head, Department of Pediatric Dentistry, Maulana Azad Institute of Dental Sciences, New Delhi, India

**Keywords:** Supernumerary premolars, Nonsyndromic, Impacted

## Abstract

The apparently morphologically normal finding of multiple supernumerary teeth in absence of an associated systemic condition or syndrome is an uncommon phenomenon. Surgical removal of supernumerary teeth is indicated if eruption of the adjacent teeth has been delayed; altered eruption, displacement of adjacent teeth is evident or pathologies such as cystic lesion and resorption of adjacent tooth have occurred. If the risks of surgery outweigh the benefits of removal, the teeth may be left *in situ *and a regular radiographic monitoring should be made.

**How to cite this article:** Gupta S, Goswami M. Asymptomatic Nonsyndromic Multiple Supernumerary Premolars. Int J Clin Pediatr Dent 2012;5(1):84-86.

## INTRODUCTION

Supernumerary teeth may be defined as any teeth or tooth substance in excess of the usual configuration of 20 deciduous and 32 permanent teeth.^1^ The most common supernumerary teeth, listed in order of frequency, are the maxillary midline supernumeraries, maxillary fourth molars, maxillary paramolars, mandibular premolars, maxillary lateral incisors, mandibular fourth molars and maxillary premolars.^2^ Over 20 syndromes and developmental conditions have been found to be associated with single supernumerary teeth and multiple supernumerary teeth developing as part of systemic conditions, such as cleidocranial dysplasia, Gardner’s syndrome, and cleft lip and palate.^3,4^ The occurrence of multiple supernumerary teeth without any associated systemic conditions or syndromes, however, is a rare phenomenon.^5^ In such cases, the mandibular premolar region is the common site of occurrence.^6^

Supernumerary premolars are ‘extra’ teeth morphologically belonging to the premolar group. Hyperdontia affecting premolars can be single (when only one supernumerary tooth is found), multiple (if several teeth are involved) or related to a syndrome such as cleidocranial dysplasia.^7^ Their occurrence can create a variety of clinical problems, such as crowding, delayed eruption, diastema, rotations, cystic lesions, and resorption of the adjacent teeth. Hence, suitable treatment after proper clinical and radiographic evaluation is essential.^8^

A case of nonsyndromic multiple supplemental supernumerary teeth in mandibular premolar region of an adolescent male patient is presented.

## CASE REPORT

**Fig. 1:**
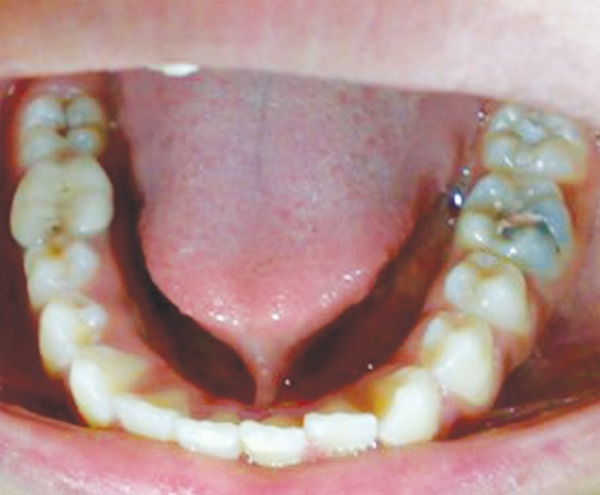
Intraoral photograph showing occlusal caries in relation to permanent left mandibular first molar

**Fig. 2:**
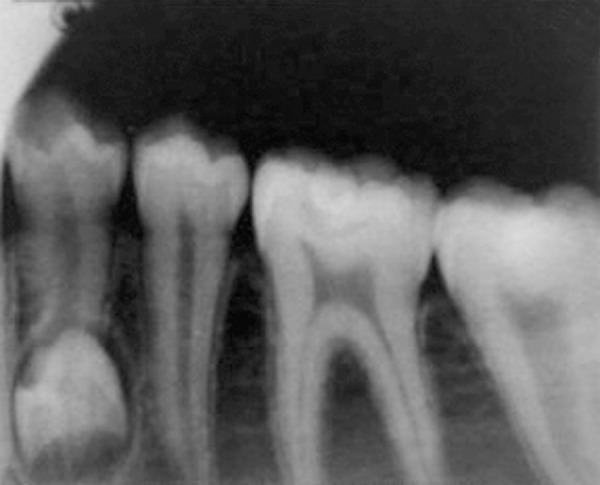
Intraoral periapical radiograph showing developing supernumerary premolar close to the root of left mandibular first premolar

**Fig. 3:**
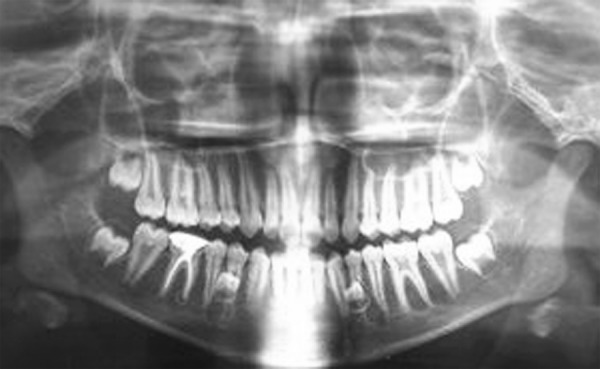
Orthopantomograph showing bilateral developing supplemental supernumerary premolars in both right and left mandibular premolar regions

**Fig. 4:**
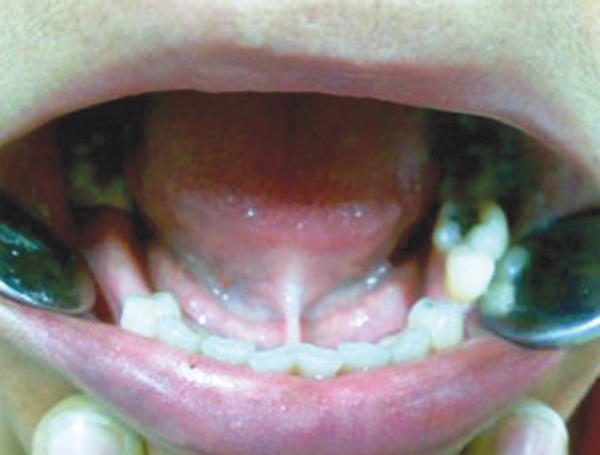
Intraoral photograph of patient’s mother showing tongue-tie

**Fig. 5:**
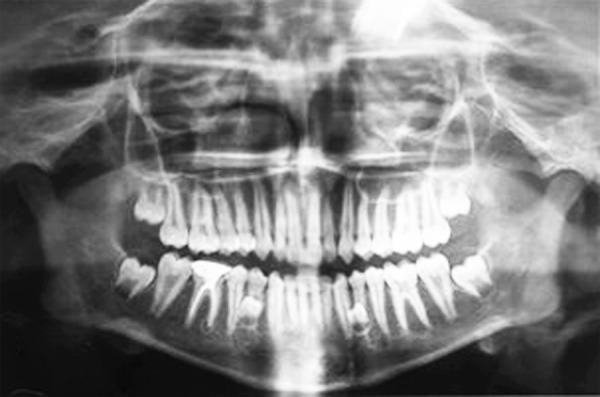
Follow-up orthopantomogram

A 13-year-old male patient presented to the Department of Pediatric Dentistry, Maulana Azad Institute of Dental Sciences, New Delhi, with a chief complaint of pain in left lower back tooth since past few days. Familial, medical and dental history was noncontributory. Extraoral examination did not reveal any abnormality. On intraoral examination, a carious lesion was present in relation to left mandibular first permanent molar; however, there was no tenderness on vertical percussion (Fig. 1). A well-aligned full complement of teeth up to second molar was present in all quadrants with fair oral hygiene. A routine intraoral periapical radiograph of the tooth was advised to assess the extent of carious involvement. The radiograph (Fig. 2) revealed the carious lesion involving dentin, but not approximating the pulp, with an associated finding of a calcified structure resembling the crown of a developing premolar close to the root of left mandibular first premolar. The calcified structure was surrounded by a radiolucent halo of the dental sac. A follow-up orthopantomograph (Fig. 3) was then advised to rule out presence of other supernumerary teeth elsewhere in the jaws. A distinct, yet similar appearing calcified structure was observed lying in its respective dental sac in relation to the root of right mandibular first premolar. The patient, however, was unaware of this and was completely asymptomatic. Hence, the left mandibular first molar was restored, while no invasive procedure was considered for the supernumerary teeth. The patient and his mother were educated about the radiographic finding and adequately counseled. The patient also had ankyloglossia though there was no restriction of tongue movements or difficulty in speech. When explained about the treatment for it, the patient’s mother was reluctant to get any surgical intervention done since she had a similar problem (Fig. 4) without any complaint. Hence, a decision was made to keep the patient on regular follow-up. After 6 months, another orthopantomograph (Fig. 5) was taken and compared with the earlier one. There was no evidence of any radiolucency or increase in size of the follicular space of the impacted teeth. The patient is still on 6-monthly follow-up.

## DISCUSSION

It has been reported that the prevalence of the supernumerary premolars in permanent dentition is between 0.075 and 0.26% and that supernumerary premolars account for only 10% of all the supernumerary cases.^9^ The difference of these teeth from the other supernumeraries is that they more commonly occur in the mandible.^6^ Single supernumeraries occur in 76 to 86% of cases, double supernumeraries occur in 12 to 23% of the cases and multiple supernumerary teeth in less than 1% of cases.^3^ The prevalence of supernumerary premolars has been reported differently in various studies due to the differences in patient population samples, age groups, ethnicity and applied radiographic techniques.^10^ In a study conducted in Mexico on 2241 patients, Salcido- García et al^11^ reported the prevalence of the premolars to be 1.7% whereas another study by Rubenstein et al,^12^ in which 1100 orthodontic patients were included, prevalence of supernumerary premolars was found to be 0.64%.

The apparently morphologically normal finding of multiple supernumerary teeth in the absence of an associated systemic condition or syndrome is an uncommon phenomenon. Yusof^6^ reviewed most of the published cases in the English literature and found that when nonsyndromal multiple supernumerary teeth are present, the most common site affected is the mandibular premolar region, followed by the molar and the anterior regions respectively.^13^

Supernumerary premolars are usually asymptomatic and most cases are diagnosed by chance during inspection of radiographs prior to the commencement of orthodontic treatment.^10^ Bodin et al^14^ have reported that only 2% of the supernumerary premolars are likely to undergo pathological changes. Nevertheless, the most commonly encountered complications with these teeth are dentigerous cyst and root resorption at the adjacent tooth.^10^ Although it has not yet been reported, compression of the supernumerary premolars on the adjacent teeth and their closeness to the mental and inferior dental nerves may lead to pain. In planning treatment alternatives for impacted supernumerary premolars, the potential risks of leaving them *in situ *and the hazards of surgical removal of these teeth especially around the lower premolar region, where the teeth are close proximity to the inferior dental and mental nerves, should be assessed judiciously.^15^

According to many clinicians, surgical removal of these teeth is the only method of treatment.^10^ The timing of surgical removal of supernumerary premolars is as much debated among clinicians as are the treatment methods. ^10^ Whenever these teeth are associated with any pathological formation or when they hinder the eruption of, or give rise to a malpositioning of the permanent teeth, they should be removed as soon as possible.^3^ However, if the risks of surgery outweigh the benefits of removal, the teeth may be left *in situ*, as in the present case, and a regular clinical and radiographic monitoring should be made.^16^ Many clinicians recommend leaving asymptomatic supernumerary premolar teeth *in situ *until development of the adjacent anatomic structures and root development of the adjacent teeth has been completed at the end of the permanent dentition.^10^

Nevertheless, patients who are reluctant to accept the proposed therapy should be educated about the potential risks and complications of this phenomenon and they should be kept under observation.

The presence of multiple supernumerary teeth is usually associated with problems of displacement, rotation, ectopic eruption of the adjacent teeth, resorption of the adjacent teeth and even the formation of primordial cysts.^5^ An unexpected finding in the documented case is that the full complement of permanent dentition was present in normal occlusion, with none of the associated potential problems. The patient was completely asymptomatic and the presence of supernumerary teeth was an incidental finding. Because the impacted supernumeraries were not associated with any cyst formation or resorption of the adjacent teeth, it was decided to observe the teeth and to radiographically review the patient.
